# Development of Abiraterone Acetate Nanocrystal Tablets to Enhance Oral Bioavailability: Formulation Optimization, Characterization, In Vitro Dissolution and Pharmacokinetic Evaluation

**DOI:** 10.3390/pharmaceutics14061134

**Published:** 2022-05-26

**Authors:** Yuanfen Liu, Yuqi Li, Pengcheng Xu, Yan Shen, Baoqiang Tang, Qiyue Wang

**Affiliations:** 1 Department of Clinical Medicine, Jiangsu Health Vocational College, Nanjing 211800, China; liuyuanfen2010@gmail.com; 2 Department of Pharmaceutics, College of Pharmacy, China Pharmaceutical University, Nanjing 211198, China; lyq2019_cpu@163.com (Y.L.); shenyan19820801@126.com (Y.S.); 3 Department of Pharmaceutical Engineering, College of Pharmacy, Inner Mongolia Medical University, Hohhot 010110, China; xpc-impu@163.com; 4 Shenzhen Aoqi Biological Medicine Co., Ltd., Shenzhen 010110, China; 5 School of Pharmaceutical Science, Nanjing Tech University, Nanjing 211816, China

**Keywords:** abiraterone acetate, nanocrystal, poloxamer, dissolution, oral bioavailability

## Abstract

Abiraterone acetate is a prodrug of abiraterone used in combination with prednisone as a standard therapeutic strategy for hormone-resistant prostate cancer (mCRPC). Due to the poor solubility and permeability, the release and absorption of abiraterone acetate are low and reduce its bioavailability. In this project, abiraterone acetate tablets prepared using nanocrystal technology were developed to overcome the drawbacks of normal tablets by enhancing in vitro dissolution rate and oral bioavailability. The abiraterone acetate nanocrystal suspensions were prepared by top-down wet milling method using a planetary ball mill with the mixture of Poloxamer 407 and Poloxamer 188 as the optimized stabilizer at a ratio of 7:1. The optimized nanocrystals were freeze-dried and characterized using DLS, TEM, DSC, and XRD. The abiraterone acetate nanocrystal tablets significantly improve the in vitro dissolution rate of abiraterone acetate compared to raw materials. Although exhibiting a similar dissolution rate compared to the Zytiga^®^ tablets, the nanocrystal tablets significantly improve the oral bioavailability with C_max_ and AUC_0–t_ being 3.51-fold and 2.80-fold higher, respectively, in the pharmacokinetic study. The present data indicate that nanocrystal is a promising strategy for improving the dissolution and bioavailability of abiraterone acetate.

## 1. Introduction

**Scheme 1:**
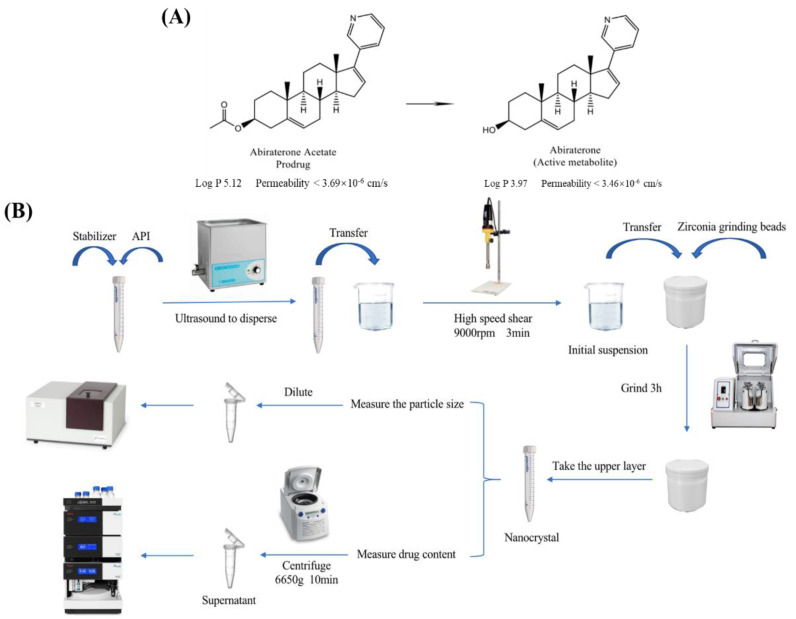
(**A**)The molecular structure of abiraterone acetate (prodrug) and abiraterone (active metabolite). (**B**) The process of preparing abiraterone acetate nanocrystals.

Prostate cancer remains the second leading cause of cancer-related death in men in the United States [1]. Androgen deprivation therapy is the basic therapeutic assay to inhibit prostate cancer growth and provide clinical benefits [2]. Commercial tablet Zytiga^®^ with the active pharmaceutical ingredient of abiraterone acetate was approved by the FDA in 2011 and is the first choice together with prednisone, a corticosteroid, for the treatment of patients with metastatic castration-resistant prostate cancer (mCRPC) and metastatic high-risk castration sensitive prostate cancer (mCSPC) [3,4,5]. As the structure shown in Scheme 1A, abiraterone acetate is a hydrophobic small molecule with a Log *p* value (the partition coefficient between octanol and water) of 5.12 [6]. Abiraterone acetate is the inactive prodrug and could be metabolite to the active state of abiraterone by the esterase. Abiraterone is a selective and irreversible inhibitor of the cytochrome P450 C17 enzyme, a key enzyme in the steroidogenic pathway, resulting in a reduction in biosynthesis and production of androgen levels in prostatic tumor tissues followed by inhibiting tumor progression [7].

The specific structure results in low solubility and permeability of abiraterone acetate, which is defined as a BCS class IV compound [8]. The absolute bioavailability of abiraterone is estimated up to 10% in the fasted state, while has a 10-fold increase in AUC and a 17-fold increase in C_max_ when administrated with food [9]. The solubility of abiraterone acetate and abiraterone in intestinal fluids is determined by the fed state and the food categories [10]. For this reason, Zytiga^®^ has to be administrated on an empty stomach, at least one hour before or at least two hours after a meal, to avoid the variable overexposures and serious side effects. Considering their poor bioavailability, abiraterone acetate daily doses are surprisingly higher to 1000 mg (four of 250 mg tablets or two of 500 mg tablets) which is not economic. Another tablet dosage form named Yonsa^®^ was prepared using abiraterone acetate particles with sizes ranging from 200 to 800 nm and exhibits a modest two-fold bioavailability improvement in humans [11]. Therefore, how to use pharmaceutical technologies to improve solubility and bioavailability is the main research direction on formulation designing for the BCS class IV drugs.

Recently, several technologies have been successfully developed to enhance the solubility, dissolution, and bioavailability of poorly soluble drugs, such as using surfactants, cyclodextrin inclusion compounds, solid dispersions, liposomes, micelles, or solid lipid nanoparticles to solubilize drugs [12,13,14,15,16,17]. Nanoparticles prepared using biodegradable copolymer (poly(DL-lactide-co-glycolide)) or lipid materials could encapsulate hydrophobic molecules inside to provide the targeted delivery and controlled release. However, lacking suitable biosafety excipients and effectively drug loading in these formulations with complicated manufacturing processes are the key obstacle the researchers need to overcome [18,19,20]. Nanocrystal technology sheds new light on advanced drug development [21]. Nanocrystals, also known as nanosuspensions, refer to a particle with a size in the nanometer range and formed by a single or multi-crystalline arrangement [22]. Drug nanocrystals are generally considered to be a carrier-free submicron colloidal dispersion system, consisting only of active pharmacological ingredients (API) and basic stabilizers, with a solid drug core surrounded by a layer of stabilizers [23,24,25]. According to the Noyes-Whitney equation and Ostwald-Freundlich equation, the nanocrystal could significantly enhance the surface area compared to raw materials leading to a huge improvement in dissolution rate [26,27]. At the same time, nanocrystals prepared with fewer types of excipients exhibit in vivo safety with good stability and are suitable for various administration routes such as oral delivery, injection delivery, ocular delivery, pulmonary delivery, and intramuscular injection [28,29,30,31,32,33]. The new technology is considered as the potential solution to effectively improve drug dissolution and absorption. Moreover, improving the dissolution and absorption by using advanced delivery systems such as nanocrystals could reduce the variability of pharmacokinetics (PK) and minimize the food effects, closer to the further goal which is ultimately removing food effects on poorly dissolution drugs [34].

In this study, abiraterone acetate nanocrystals were prepared via a rapid top-down wet milling method using a planetary ball mill. We optimized the preparation references and stabilizers in the formulation to develop freeze-dried nanocrystals. We further characterized the freeze-dried nanocrystals and investigated the in vitro dissolution, and in vivo pharmacokinetic performance of abiraterone acetate nanocrystal tablets to improve abiraterone bioavailability.

## 2. Materials and Methods

### 2.1. Materials

Abiraterone acetate (purity ≥ 99.0%) was purchased from Hubei Zhonglong Kangsheng Fine Chemical Co., Ltd. (Wuhan, China). Zytiga^®^ reference tablets were purchased from the Zhongda Hospital Affiliated with Southeast University (Nanjing, China). Phenacetin standard product (purity 98.7%) was purchased from Dr. Ehrenstorfer™ (Shanghai, China). Abiraterone standard product and formic acid (HPLC grade, 99.9%) both were purchased from Aladdin (Shanghai, China). Poloxamer 407 (P407), Poloxamer 188 (P188), Povidone K30 (PVPK30), and sodium dodecyl sulfate (SDS) were all purchased from BASF (Shanghai, China). Hydroxypropyl methylcellulose (HPMC) was obtained as a gift from Anhui Sunhere Pharmaceutical Excipients Co., Ltd. (Huainan, China). Magnesium stearate and croscarmellose sodium were obtained as a gift from Fenghong Pharmaceutical Excipients Technology Co., Ltd. (Shanghai, China). Microcrystalline cellulose PH102 was the purchase of Asahi Kasei Chemicals Corporation (Guangzhou, China). Lactose was obtained as a gift from MEGGLE (Shanghai, China). Acetonitrile and Methanol (HPLC grade) were purchased from TEDIA (Fairfield, OH, USA).

Male Wistar rats weighing 230–270 g were purchased from Biocloon Co., Ltd., Nanjing (Nanjing, China). All the procedures involving animals were approved by the Institutional Animal Care and Use Committee (IACUC) of China Pharmaceutical University and meet all the requirements of animal experimentation to ensure proper care and use of laboratory animals for research (No. 2021-005-03).

### 2.2. Preparation of Freeze-Dried Abiraterone Acetate Nanocrystals

The process of preparing abiraterone acetate nanocrystals was as follows in Scheme 1B. The nanocrystals were prepared by a planetary ball mill (YXQM-1L, MITR, Changsha, China) using 60 g of grinding beads (ø 0.2–0.4 mm, zirconium oxide) milling with 1.5 g of abiraterone acetate, 262.5 mg of P407, 37.5 mg of P188, and 15 mL of deionized water. The stabilizer and APIs were mixed by the ultrasound and high-speed shear at 9000 rpm for 3 min to form the initial suspension. The nanocrystal suspension was ground with the revolution speed of 150 r/min, auto-transmission speed of 300 r/min, and grinding time of 180 min. During the running process, the forward and reverse directions were alternated every 5 min to obtain a unit size distribution. At last, the nanocrystals were separated from the milling pearls for further investigation.

Although stabilizers in formulation could help to maintain the stability of nanocrystals, the thermodynamics and molecular dynamics behavior in the liquid phase is more intense than in the solid phase, which still accelerates the nanocrystal agglomeration and cluster growth. Therefore, dispersed nanocrystals need to be dry to maintain their stability. An appropriate amount of lyophilization protector was mixed with products to prepare the freeze-dried abiraterone acetate nanocrystal powder.

### 2.3. Optimization of Formulation and Preparation Process

To optimize the formulation of nanocrystals, a series of single-factor experiments were designed to investigate: (1) the type of stabilizer (HPMC, P407, P188, PVPK30, and SDS) and (2) the mass ratio of the stabilizer to abiraterone acetate (1:5 and 1:2). Moreover, the preparing references were also optimized to obtain the freeze-dried nanocrystals with suitable physicochemical properties such as the rotating speed of the planetary ball mill (90 r/min and 150 r/min), the type of lyophilization protector (lactose, trehalose), the amount of lyophilization protector (2%, 5%, and 10%), and the ratio of mixed stabilizer (9:1 to 5:5). The particle size, polydispersity index (PDI), and drug content were taken as evaluation indicators to determine the optimal formulation of abiraterone acetate nanocrystals.

### 2.4. Characterization of Freeze-Dried Abiraterone Acetate Nanocrystals

#### 2.4.1. Transmission Electron Microscopy (TEM)

Morphology of abiraterone acetate nanocrystals was carried out with a transmission electron microscope (TEM, Ht7700, HITACHI, Tokyo, Japan). A number of freeze-dried abiraterone acetate nanocrystals were dispersed in distilled water by ultrasonic for 3 min and deposited onto a copper mesh with holey carbon film. The phosphotungstic acid aqueous solution 1% (*w*/*v*) was used to negatively stain the copper mesh. After drying, the copper mesh was observed under an electron microscope under an operating voltage of 200 kV for TEM characterization.

#### 2.4.2. Particle Size and Zeta Potential

The particle size, PDI, and zeta potential of abiraterone acetate nanocrystals were measured by dynamic light scattering (DLS) assay using a laser particle size distribution analyzer (NanoBrook Omni, Brookhaven Instruments, Holtsville, NY, USA). Freeze-dried abiraterone acetate nanocrystals were dispersed in distilled water and diluted to an appropriate concentration before measurement. Each sample was performed in triplicate at a fixed angle of 90° at 25 °C.

#### 2.4.3. Differential Scanning Calorimetry (DSC)

Thermal phase transitions of the nanocrystal samples were analyzed using differential scanning calorimetry (DSC250, TA, New Castle, DE, USA). Abiraterone acetate, a physical mixture with lyophilization protector, and the freeze-dried abiraterone acetate nanocrystals (about 5 mg for each sample) were carefully transferred into an aluminum pan sealed with a perforated lid. DSC was performed with a heating rate of 5 °C/min with a heating range from 20 °C to 200 °C and the transition curve of samples was recorded. Nitrogen purge with a rate of 50 mL/min was used during the whole measurement process.

#### 2.4.4. Powder X-ray Diffraction (XRD)

X-ray powder diffraction patterns of abiraterone acetate and freeze-dried abiraterone acetate nanocrystals were measured using a D8 Advance X-ray powder diffractometer (Bruker, Germany). Samples were loaded onto a sample holder and smoothed the surface using a blade. Samples were analyzed with the 2*θ* range from 0–60° at a scanning speed of 4°/min and a step size of 0.02°.

#### 2.4.5. Determination of Abiraterone Acetate Content

The abiraterone acetate concentration in nanocrystals was determined by high-performance liquid chromatography (HPLC) (Thermo Fisher Scientific, Ultimate 3000, Waltham, MA, USA) performed on Ultimate^®^ XB-C18 chromatographic column (250 mm × 4.6 mm, 5 μm) with a mobile phase of methanol-water (95/5, *v*/*v*) at a flow rate of 1.0 mL/min and injection volume of 20 μL. The detection wavelength and column temperatures were 254 nm and 40 °C, respectively.

### 2.5. Preparation of Abiraterone Acetate Nanocrystal Tablets

The abiraterone acetate nanocrystal tablets were prepared by the dry granulation method. Briefly, freeze-dried abiraterone acetate nanocrystals, fillers (lactose, microcrystalline cellulose), and disintegrants (croscarmellose sodium, PVPK30, and colloidal silicon dioxide) were accurately weighed and mixed followed by compressed into flakes. The flakes were re-grinded and passed through mesh 50^#^ (with a hole diameter of 0.32 mm). Then, magnesium stearate as a lubricant was added at last and mixed uniformly by passing through mesh 50^#^ again. A rotary tableting machine (MINI Press I-16, Karnavati Engineering, Gujarat, India) was used to press the formulation mixture to prepare the tablets. The weight of each tablet was 715 mg with 250 mg of API and the hardness of each tablet was controlled between 80–90 N by a hardness tester. Abiraterone acetate content was accurately quantified by HPLC.

### 2.6. In Vitro Release Study

The paddle method was recommended to measure the release of abiraterone acetate tablets with the rotating speed of 50 rpm at 37 ± 0.5 °C in a dissolution apparatus (TIANDA TIANFA Pharmaceutical Testing Instrument Manufacturer, RC806D, Tianjin, China). Briefly, freeze-dried abiraterone acetate nanocrystals, Zytiga^®^ reference tablets, and abiraterone acetate nanocrystal tablets were directly put into the dissolution vessel with a 900 mL dissolution medium which was prepared by pH 4.5 phosphate buffer (56.5 mM) containing 0.25% (*w*/*v*) of SDS. During dissolution, 10 mL of release medium was collected at certain time intervals (5, 15, 30, 45, and 60 min) after the experiment started and an equivalent volume of fresh medium was supplemented immediately. The collected sample was analyzed by HPLC and data were used to calculate the cumulative dissolution rate and dissolution curve. To investigate the similarity of the dissolution behavior of reference and abiraterone acetate nanocrystal tablets in vitro, the similarity factor of the dissolution profile (f_2_) was calculated.

Cumulative dissolution (%) is calculated according to the following equation:

$$\mathrm{Cumulative}\;\mathrm{dissolution}=[\frac{C_{n}}{L/V_{2}}+\frac{V_{1}\times \frac{\left(C_{n-1}+\ldots C_{2}+C_{1}\right)}{L/V_{2}}}{V_{2}}]\times 100\%$$

where *C_n_* represents sample concentration after removal of every point in time, *L* represents the labeled amount of preparation, *V*_1_ represents the sampling volume of every point in time, and *V*_2_ represents the volume of dissolution media.

The formula of f_2_ is according to the following equation:

$$f_{2}=50\times \log\;\{[1+\;\frac{1\;}{\;n}\sum_{t=1}^{n}{\left(R_{t}-T_{t}\right)}^{2}{]}^{-0.5}\times 100\}\;$$

where *R_t_* represents the average cumulative release of the reference formulation at the *t* time point, *T_t_* represents the average cumulative release of the test formulation at the *t* the time point, and *n* represents the number of test points.

### 2.7. In Vivo Pharmacokinetic Assay

In vivo pharmacokinetic studies were performed on two groups of male Wistar rats (*n* = 6) weighing 230–270 g and the rats fasted for 12 h with free drinking before administration. The rats were administered with the suspension of reference and abiraterone acetate nanocrystal tablets by gavage at a single dose of 100 mg/kg. Blood samples were collected from the retro-orbital venous plexus at certain time intervals (15, 30, 60, 120, 240, 480, 720, and 1440 min) post-administration, respectively. Blood samples were centrifuged for 5 min at 3000 rpm to collect plasma and stored at −20 °C for further analysis.

In total, 12.5 µL of IS solution (10 µg/mL phenacetin in acetonitrile) and 25 µL of plasma samples were mixed by vortex for 30 s followed by mixing with 87.5 µL of a methanol solution containing 0.1% formic acid for 30 s to precipitate the plasma proteins. After centrifugation (10,000 rpm, 10 min, room temperature), 150 µL of the supernatant was transferred and mixed with 50 µL of 50% methanol aqueous solution which was added to dilute, and was then analyzed using LC-MS/MS (LC-MS 8050, SHIMADZU, Kyoto, Japan). The mass spectrometer will be operated in the positively selected reaction monitoring (SRM) for abiraterone acetate (*m*/*z* 392.15/330.30), abiraterone (*m*/*z* 352.30/157.15), and internal standard (*m*/*z* 180.15/110.15). Shimadzu LC-20 AT equipped with Poroshell 120 SB-C18 Column (2.7 µm, 2.1 mm × 100 mm (Waters Corp)) was used for compound separation. Analyte separation (abiraterone acetate, abiraterone, and phenacetin) was carried out using a gradient elution including mobile phase A (0.1% formic acid in water) and mobile phase B (0.1% formic acid in acetonitrile) delivered at 0.6 mL/min. The time program of the gradient method was set as mobile phase B 95% (2 min), 10% (2.1–4 min), and 95% (4.1–6 min). LC-MS/MS data acquisition will be performed by using software on the mass spectrometer. LC/MS/MS data acquisition was performed by using LC SOLUTION software.

Pharmacokinetic parameters were calculated from experimental data by a non-compartmental model using WinNonlin software, including the area under the plasma concentration–time curve (AUC), the maximum plasma drug concentration (C_max_), the time to reach C_max_ (T_max_), and the relative bioavailability (F_rel_).

### 2.8. Statistical Analysis

Comparisons between two different groups were analyzed by Student’s *t*-test, and one-way ANOVA was performed for three or more groups. All obtained data were expressed as the mean ± SD, and significant differences between or among groups are indicated by * *p <* 0.1, ** *p <* 0.05.

## 3. Results and Discussion

### 3.1. Optimization of Formulation and Preparation Process

#### 3.1.1. The Mass Ratio of the Stabilizer to Drug

**Figure 1:**
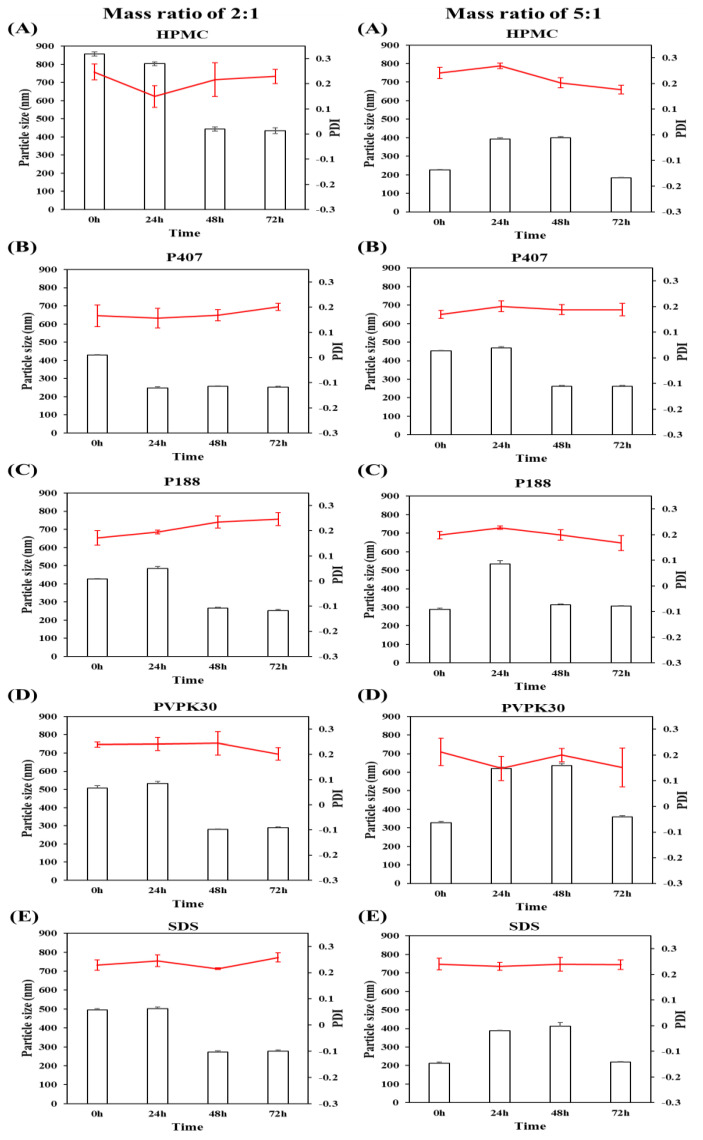
The particle size (columns) and PDI (red lines) of abiraterone acetate nanocrystals prepared with the mass ratio of the drug to different stabilizers were 2:1 or 5:1 and stored at 4 °C for 72 h (*n* = 3). (**A**) HPMC, (**B**) P407, (**C**) P188, (**D**) PVPK30, (**E**) SDS. The concentration of the drug was 1 mg/mL.

To optimize the amount of the stabilizer for nanocrystal preparation, we investigated the mass ratio of the drug to the stabilizer at 2:1 and 5:1 using different types of stabilizers and using particle size as the determinants. As shown in Figure 1, the particle size of nanocrystals was significantly affected by the mass ratio of the stabilizer to the drug no matter the type of surfactant, indicating it is a factor more important. With a stabilizer-drug ratio of 1:5, the particle size of nanocrystals could be stable at around 200–400 nm and keep stable for at least 72 h. The stability of the particles (including nanocrystals) is based on the efficiency of the collision. With the suitable stabilizer concentration, collisions between particles decreased due to a reduction in collision efficiency and therefore improved their short-term stability. While increasing the amount of stabilizer to the ratio of 1:2 surprisingly increased the particle size of nanocrystals. We speculate that an overdose of surfactant could also enhance particle agglomeration and sedimentation owing to excessive adsorption. While the hydrophobic core of polymers attaches to the surface of the particle, leaving its hydrophilic party dispersing in the solution [35]. The overdose stabilizer with a concentration much higher than critical micelle concentration (CMC) no longer exhibits spherical micelles but might the layered or plate structure with helped nanocrystal accumulation. Therefore, the stabilizer-drug ratio of 1:5 was chosen as the optimized formulation of the abiraterone acetate nanocrystals.

#### 3.1.2. The Type of Stabilizers

**Figure 2:**
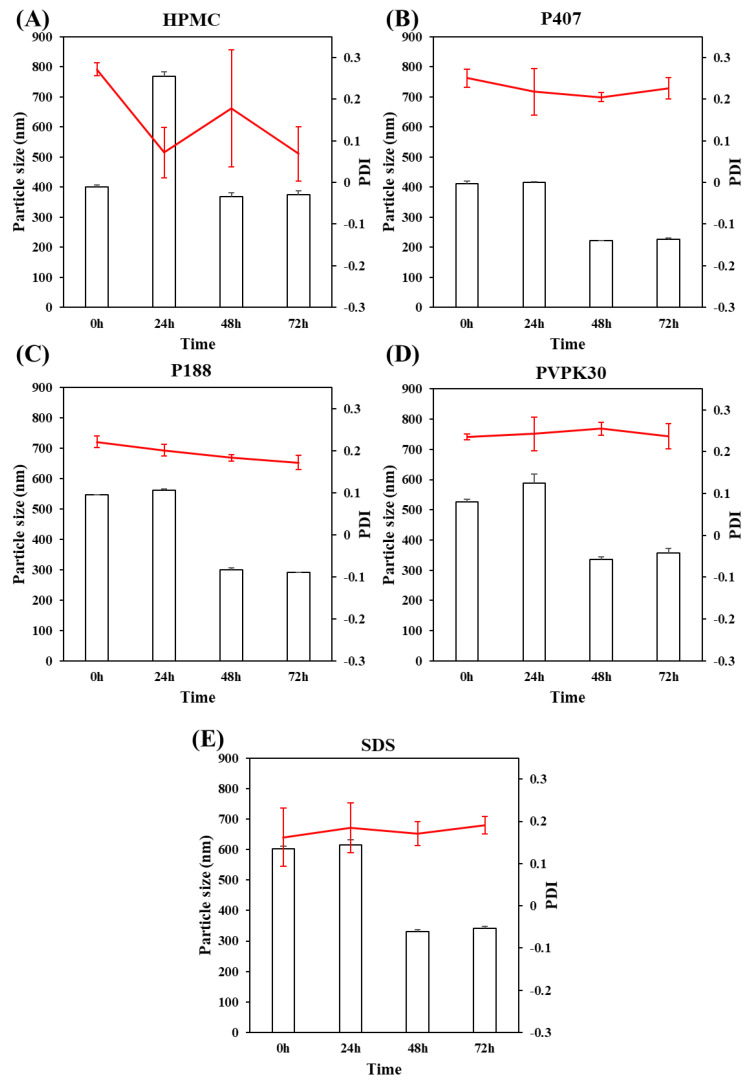
The particle size (columns) and PDI (red lines) of abiraterone acetate nanocrystals prepared with the mass ratio of the drug to different stabilizers were 5:1 and stored at 4 °C for 72 h (*n* = 3). (**A**) HPMC, (**B**) P407, (**C**) P188, (**D**) PVPK30, (**E**) SDS. The concentration of the drug was 2 mg/mL.

To investigate the effects of types of stabilizers on the particle size of the nanocrystal, we firstly use HPMC, P407, P188, PVPK30, and SDS alone to prepare the nanocrystal. Figure 1 and Figure 2 exhibits the effect of set time and drug concentration on the particle size and PDI of abiraterone acetate nanocrystals, respectively. Using HPMC or PVPK30 as the stabilizer, the PDI of nanocrystals changed evidently with the elongation of the set time which hard to keep uniform and stable. Furthermore, the particle size of nanocrystals using SDS as the stabilizer was significantly increased with the increase in drug concentration from 1 mg/mL to 2 mg/mL. Abiraterone acetate nanocrystals prepared with P407 and P188 as stabilizers could maintain the particle size and PDI at the different set times and drug concentrations making them suitable for the formulation preparation.

A good stabilizer should meet two conditions: firstly, it should be able to be tightly adsorbed on the surface of nanocrystals, and there should be strong intermolecular interactions between the stabilizer and the drug, among which the hydrophobic interaction is dominant, and if there are strong interactions such as hydrogen bonds, the stabilization effect is better; the second is to have a suitable hydrophilic-lipophilic balance value, because the hydrophobicity is too strong, although it can be tightly adsorbed on the surface of nanocrystals, poor hydrophilicity will lead to higher surface energy, and the stabilization effect is not good. Ideally, there should be a moderate balance of hydrophilic and lipophilic. With the decrease in the particle size, the specific surface area and surface energy of the particles exponentially increase. Large particles will adsorb small particles to cause sedimentation and the settled particles cannot be re-dissolved based on the Oswald ripening mechanism. However, the remaining smaller particles will maintain the smaller size with the elongation of the set time.

**Figure 3:**
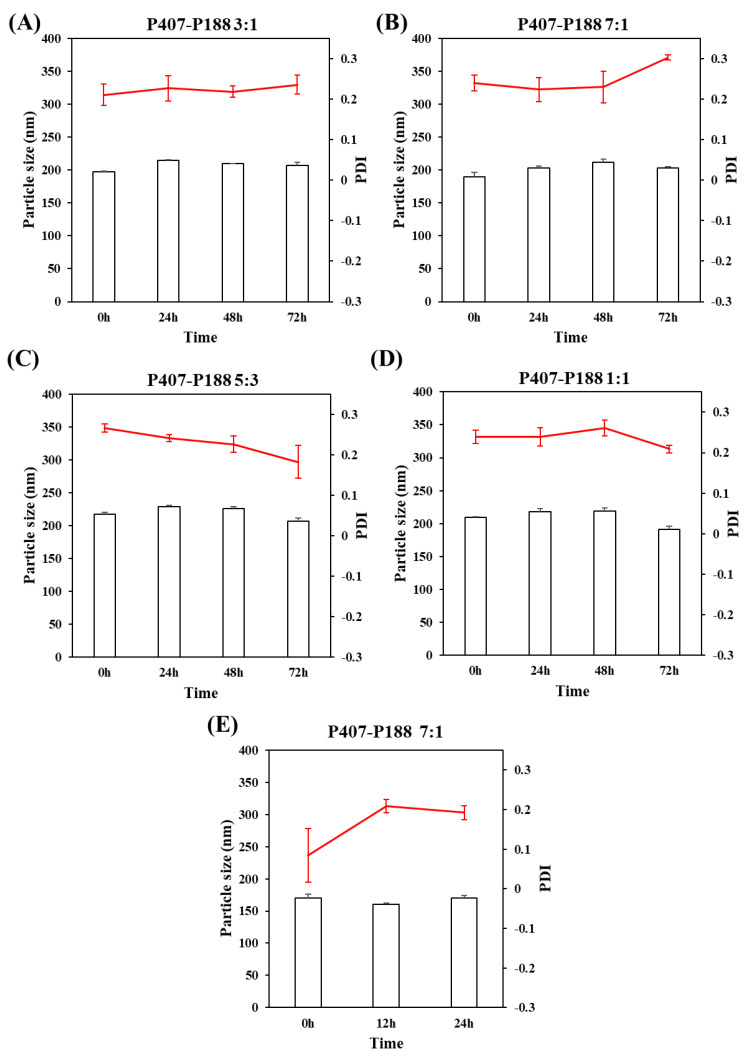
The particle size (columns) and PDI (red lines) of abiraterone acetate nanocrystals were prepared with the co-stabilizer of P408 and P188 at different mass ratios: (**A**) 3:1; (**B**) 7:1; (**C**) 5:3; (**D**) 1:1 (*n* = 3). The concentration of the drug was 2 mg/mL. (**E**) The particle size and PDI of nanocrystals placed at 4 °C for 24 h with a co-stabilizer of P407 and P188 at a drug concentration of 30 mg/mL (*n* = 3).

Related literature reports that P407 formulations enhance the solubilization of poorly water-soluble drugs, but it does not clearly show any related advantages when used alone [36]. The combination with other excipients such as P188 or mucoadhesive polymers promotes the sol–gel transition behavior, solubilizing ability, or bioadhesive properties compared to using P407 alone. Therefore, we investigated the possibility of using P407 and P188 as co-stabilizer to prepare nanocrystals. According to Figure 3A–D, the particle size of the nanocrystals was universally about 200 nm with various ratios of P407:P188 from 7:1 to 1:1, which is significantly smaller than that prepared by a single stabilizer. Moreover, as shown in Figure 3E, the particle size keeps stable within 72 h with the drug concentration increase to 30 mg/mL, indicating that the prepared nanocrystals with these combined stabilizers had better stability. The abiraterone acetate nanocrystals prepared with P407 and P188 as the stabilizers at a mass ratio of 7:1 had the highest entrapment efficiency (48.3% ± 0.7%) with a zeta potential of −57.85 mV ± 0.194 mV. In addition, the particle size and PDI were not affected by the increase in drug concentration and the elongation of time.

#### 3.1.3. Effect on the Rotating Speed of Planetary Ball Mill

**Figure 4:**
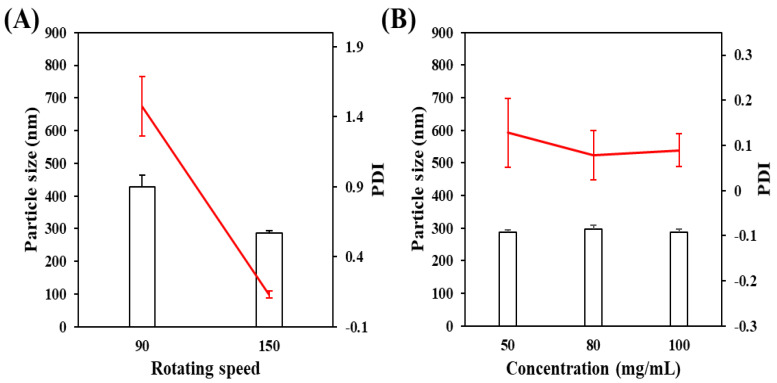
The particle size (columns) and PDI (red lines) nanocrystals were prepared at the different planetary ball mill (**A**) rotating speeds and (**B**) drug concentrations (*n* = 3).

The preparation process also strongly affects the physicochemical property. As shown in Figure 4A, the particle size and PDI of abiraterone acetate nanocrystals were dependent on the rotating speed of the planetary ball mill. With the increase in rotating speed, the particle size and PDI were reduced significantly. The stability of nanocrystals is affected by the temperature increase caused by higher rotating speeds. Considering the bearing capacity of the planetary ball mill, the higher revolution speed was not investigated anymore and the revolution speed was set to 150 r/min during the preparation process. In addition, the concentration of nanocrystals has no obvious effect on the particle size of abiraterone acetate nanocrystals (Figure 4B).

#### 3.1.4. The Type of Lyophilization Protector

**Figure 5:**
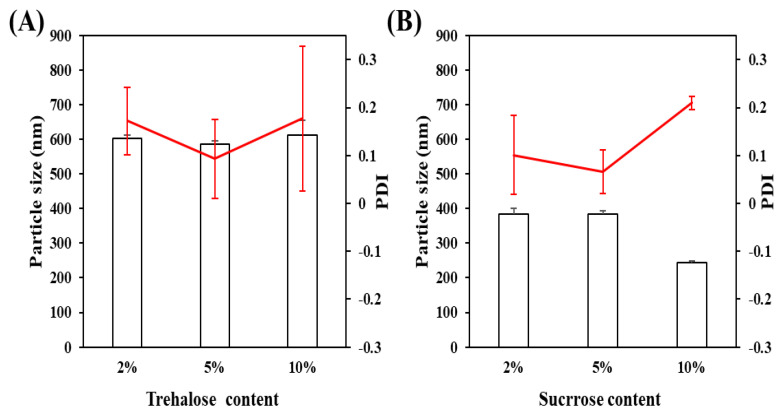
The particle size (columns) and PDI (red lines) of nanocrystals after re-dispersion formulating with lyophilization protectors: (**A**) trehalose or (**B**) sucrose (*n* = 3).

The suitable lyophilization protector was used to protect the shape of abiraterone acetate nanocrystals, avoid aggregation during the storage, and improve re-dispersion. Lactose was firstly selected as a common protector based on the earlier investigation after extensive screening but belongs to reducing sugar. Subsequently, trehalose and sucrose were also found to exhibit outstanding protective effects [37]. Therefore, we investigate the type of lyophilization protectors between trehalose and sucrose and optimize the ratio in the formulation of freeze-dried abiraterone acetate nanocrystals. As shown in Figure 5, compared with the nanocrystals before freeze-drying, the particle size and PDI were increased obviously when using trehalose as the lyophilization protectors. However, the formulation containing sucrose will not affect the particle size significantly before and after the freeze-dry, indicating it is more suitable as the lyophilization protector of abiraterone acetate nanocrystals. In addition, with the increased content of protective agents, the particle size has not significantly changed compared to nanocrystals alone. The number of hydrogen bonds formed by the protective agent and abiraterone acetate molecules is beneficial to maintaining stability. Trehalose with a large molecular weight with greater steric hindrance shows a poor ability to form stable hydrogen bonds with abiraterone acetate molecules than sucrose.

Moreover, we investigate the mixture of lactose and sucrose as lyophilization protectors and optimized their mass ratio. The mass ratios of lactose and sucrose were investigated. Experimental results (data not shown) indicate that the morphology, reconstitution time, and drug content of freeze-dried abiraterone acetate nanocrystals prepared with lactose and sucrose as protective agents exhibit the best property at a mass ratio of 1:1.

### 3.2. Characterization of Freeze-Dried Abiraterone Acetate Nanocrystals

**Figure 6:**
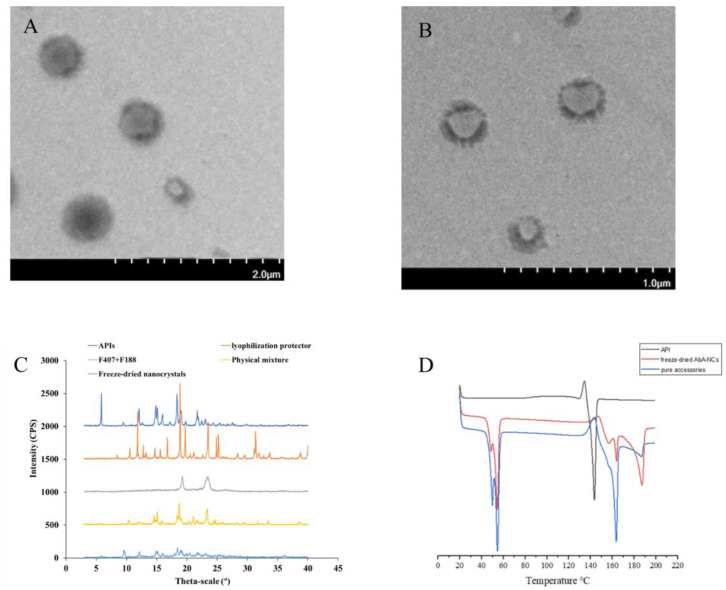
(**A**,**B**) TEM; (**C**) XRD; (**D**) DSC of freeze-dried abiraterone acetate nanocrystals.

Based on the single factor optimization, we determined the key parameters of formulation content and preparation process and prepare the freeze-dried abiraterone acetate nanocrystals for further research. Figure 6A,B (Raw images shown in Supplementary) exhibit the TEM images of nanocrystals in different fields of view to confirm their morphology. The abiraterone acetate nanocrystals form a spherical shape with a stabilizer coated outside. According to TEM, the particle size of the nanocrystals ranges from 200 nm to 500 nm, which is similar to the value measured by DLS even though there are differences in the measurement principles of DLS. This because pure mechanical grinding cannot form a completely uniform system, leading to the large size distribution observed in TEM.

Figure 6C illustrates the XRD of abiraterone acetate raw materials and freeze-dried abiraterone acetate nanocrystals. The abiraterone acetate nanocrystals exhibit the sharp diffraction peaks at 2θ of 6.1°, 12.1°, 14.8°, 15.1°, 15.9°, 18.3°, and 21.6° which are the characteristic peaks of the crystal (Raw data shown in Supplementary). The freeze-dried nanocrystals maintained all the diffraction peaks above, indicating the milling and freeze-drying process will not change the crystal from the raw drugs. However, the peak height at 6.1° significantly decreased in nanocrystals which might cause the grain size in raw drugs to decrease due to milling. Moreover, there are characteristic wide peaks of P407 and P188 which are submerged in other peaks and are hard to observe.

DSC analysis was performed on API, freeze-dried abiraterone acetate nanocrystals, and excipients alone, respectively. As shown in Figure 6D, API has an obvious melting peak at 145 °C, while freeze-dried abiraterone acetate nanocrystals produced a relatively sharp but slightly shifted melting peak at 155 °C. The shift in the melting peak position is caused by the change in particle size after grinding and because the excipients affected the melting point of the API. The freeze-dried abiraterone acetate nanocrystals and pure excipients have sharp melting peaks at 50 °C, 55 °C, 165 °C, and 190 °C, respectively, corresponding to the melting temperature of P407, P188, mannitol, and sucrose.

### 3.3. Preparation of Abiraterone Acetate Nanocrystal Tablets and In Vitro Release

The abiraterone acetate nanocrystal tablets were prepared by using the method of dry granulation. Excipients and freeze-dried abiraterone acetate nanocrystals were mixed and passed through mesh 50^#^ before compressing. The formulation was as follows in Table 1.

**Figure 7:**
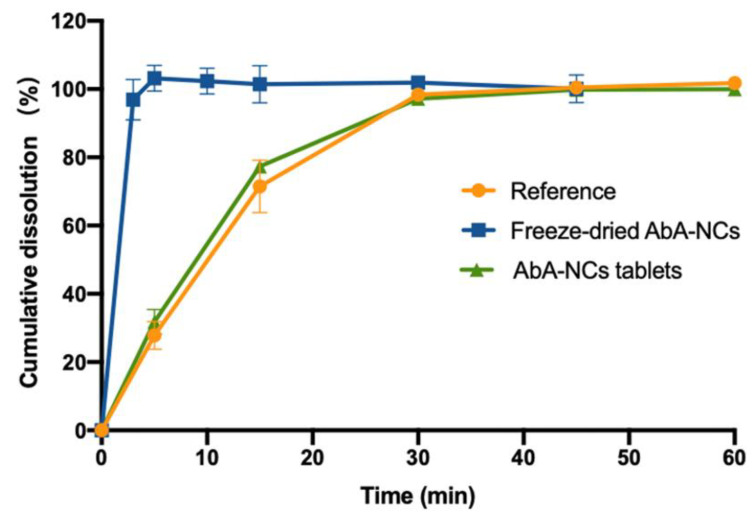
The dissolution profile of the reference, freeze-dried nanocrystals, and abiraterone acetate nanocrystal tablets with pH 4.5 at 37 °C.

The release curve clearly illustrated that the dissolution of freeze-dried abiraterone acetate nanocrystals was significantly faster than the Zytiga^®^ reference tablet. All three groups completed the release behavior of the drug within 30 min (Figure 7). The freeze-dried abiraterone acetate nanocrystals released complete drug amounts almost immediately in 3 min which is significantly faster than the raw drugs. Due to the addition of some water-insoluble excipients and the influence of pressure during preparation, the prepared abiraterone acetate nanocrystal tablets experienced the process of disintegration before they began to release. The release rate of abiraterone acetate nanocrystal tablets was slower than that of nanocrystals while similar to the reference at the same period. In addition, the f_2_ factor between the dissolution curves of the abiraterone acetate nanocrystal tablets and reference formulations was 83.26. The results show that the dissolution of the reference and abiraterone acetate nanocrystal tablets are highly similar in vitro.

### 3.4. In Vivo Pharmacokinetic Study

**Figure 8:**
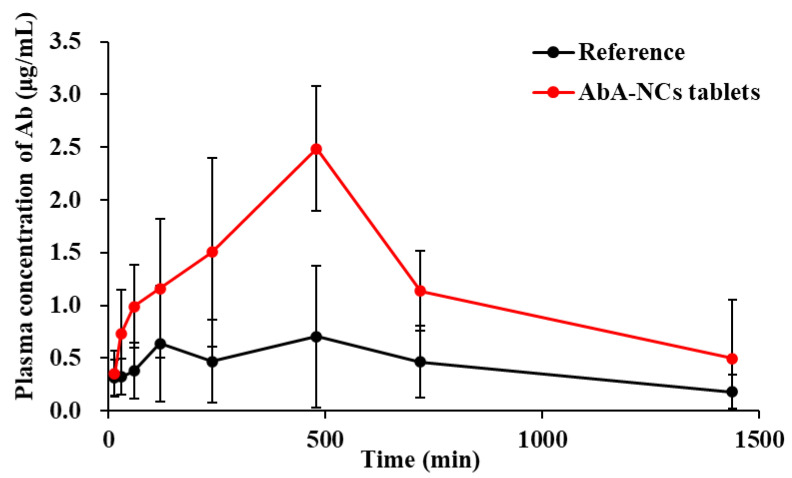
Drug–time curves of reference and abiraterone acetate nanocrystal tablets after oral administration.

Abiraterone acetate is a lactone prodrug and can be rapidly metabolized to the active metabolite abiraterone after oral administration. A summary of the pharmacokinetic parameters was provided in Table 2. The research results show that abiraterone acetate nanocrystal tablets were absorbed rapidly in vivo, which was consistent with the increase in the dissolution rate in vitro. The C_max_ of abiraterone acetate nanocrystal tablets was increased 3.5-fold, and the oral bioavailability was enhanced 2.8-fold compared with the reference tablets. There were two peaks of concentration in the pharmacokinetic profile of oral administration of the reference, which might be because of the pH changes in the digestive tract, which the absorption rate of abiraterone in different parts of the gastrointestinal tract is different (Figure 8). This study proved that the bioavailability of abiraterone is significantly improved after being dispersed to the nanocrystals.

The high bioavailability of nanocrystals may be attributed to the diverse absorption methods of nanoparticles through the gastrointestinal tract, the size of nanocrystals, and the effect of surfactants. Although the dissolution profile between the Zytiga^®^ reference tablet and abiraterone acetate nanocrystal tablets in vitro is similar, compared with micron-sized drug particles, nanocrystals can be absorbed in a variety of ways. After the drug is dissolved from nanocrystals, it can be directly absorbed across the membrane through passive transport in the molecular state. At the same time, it can also be transported and absorbed through the lymphatic system through the Pey’s node on the surface of the intestine in the form of nanocrystals. Moreover, it can be absorbed through cell-to-cell pathways or trans-cell transport [26]. Secondly, nanocrystals have general mucosal adhesion to biological mucosa including gastrointestinal tract (GIT) mucosa. Due to small the particle size, nanocrystals can quickly penetrate the gel pores of the mucus layer on the surface of the gastrointestinal tract and adhere tightly to it, thus the concentration gradient is increased and the retention time is prolonged. In addition, due to the size of the nanocrystals, they can to be effectively absorbed in the intestine, which can bypass the first-pass effect and reduce the clearance rate [38]. Meanwhile, the solubility and dissolution rate of the drug in the digestive fluid is increased by reducing the particle size to the nanoscale, so a higher drug concentration gradient is generated between the GIT and the blood vessel, which will significantly improve the absorption of the drug and increase the bioavailability. Furthermore, surfactants, such as P407 and P188, can increase the permeability of the gastrointestinal mucosa and improve the affinity and adhesion between the nanoparticles and the intestinal membrane [39].

## 4. Conclusions

The formulation of drug nanocrystals has been considered as one of the most promising routes for improving the pharmaceutical behaviors of water-insoluble APIs. In this study, abiraterone acetate nanocrystals were optimized through single factor experiments and the optimized formulation was characterized to confirm the properties. The stability of nanocrystal suspensions is the key factor in nanocrystal preparations. The stability of nanocrystals is influenced by various factors such as the types and amount of stabilizers, the process parameters, and the lyophilization protector. Therefore, optimization research is a necessary process for the successful development of a dosage form. The abiraterone acetate nanocrystals prepared with the optimal prescription were further freeze-dried and compressed to the nanocrystal tablets using the dry granulation method. The dissolution rate of abiraterone acetate nanocrystal tablets was similar to the Zytiga^®^ reference tablet in vitro, whereas the oral bioavailability in vivo was increased by 2.80-fold, indicating that nanocrystals can effectively improve the oral absorption of insoluble drugs. This shows that the abiraterone acetate nanocrystal tablet has a good application prospect and provides a new strategy for the treatment of prostate cancer. Moreover, nanocrystals with high drug loading could significantly reduce the volume of dosage forms which also provides a potential for application in other dosage forms such as injection or inhalation, even though the key obstacles on stability and re-dispersibility still need to be solved.

## Figures and Tables

**Table 1:** The formulation of abiraterone acetate nanocrystal tablets.

Element	Weight (mg)
freeze-dried abiraterone acetate nanocrystals	301.2
lactose	198.65
microcrystalline cellulose	118.62
croscarmellose sodium	42.9
povidone K30	35.75
colloidal silicon dioxide	7.15
magnesium stearate	10.73

**Table 2:** Pharmacokinetic parameters of abiraterone after oral administration of reference and abiraterone acetate nanocrystal tablets.

Parameters	Zytiga^®^ Reference Tablet	Abiraterone Acetate Nanocrystal Tablets
T_max_ (min)	480	480
C_max_ (µg/mL)	0.71 ± 0.71	2.49 ± 0.77 **
AUC_0→t_ (µg/mL × min)	629.84 ± 594.86	1763.03 ± 830.94 *
AUC_inf_ (µg/mL × min)	760.08 ± 594.13	2081.27 ± 1292.88 *
F_rel_ (%)	100	279.92

Notes: All the values are represented as mean ± SD. * *p* < 0.1, ** *p* < 0.05 compared with reference.

## Data Availability

Not applicable.

## Supplementary Materials

The following supporting information can be downloaded at: https://www.mdpi.com/article/10.3390/pharmaceutics14061134/s1, TEM images and XRD raw data.
