# Supplementary figures and images for: Development of Abiraterone Acetate Nanocrystal Tablets to Enhance Oral Bioavailability: Formulation Optimization, Characterization, In Vitro Dissolution and Pharmacokinetic Evaluation
Source: Pharmaceutics. 2022 May 26;14(6):1134. doi: 10.3390/pharmaceutics14061134 (PMC9228621; doi:10.3390/pharmaceutics14061134)

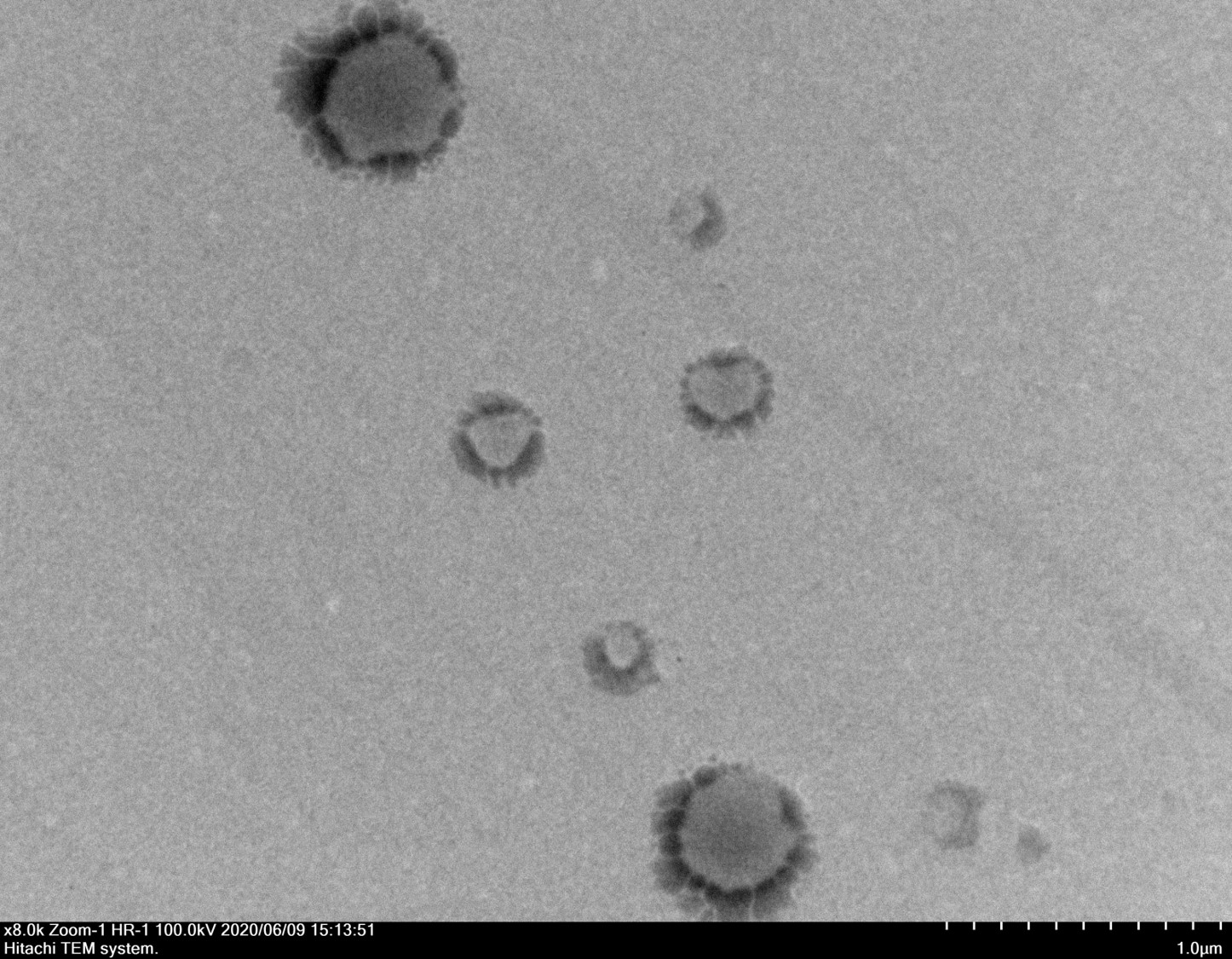

Supplement: Supplementary file 1 [file pharmaceutics-14-01134-s001.zip › pharmaceutics-1721084-supplementary/Supplementary/TEM/1.jpg]

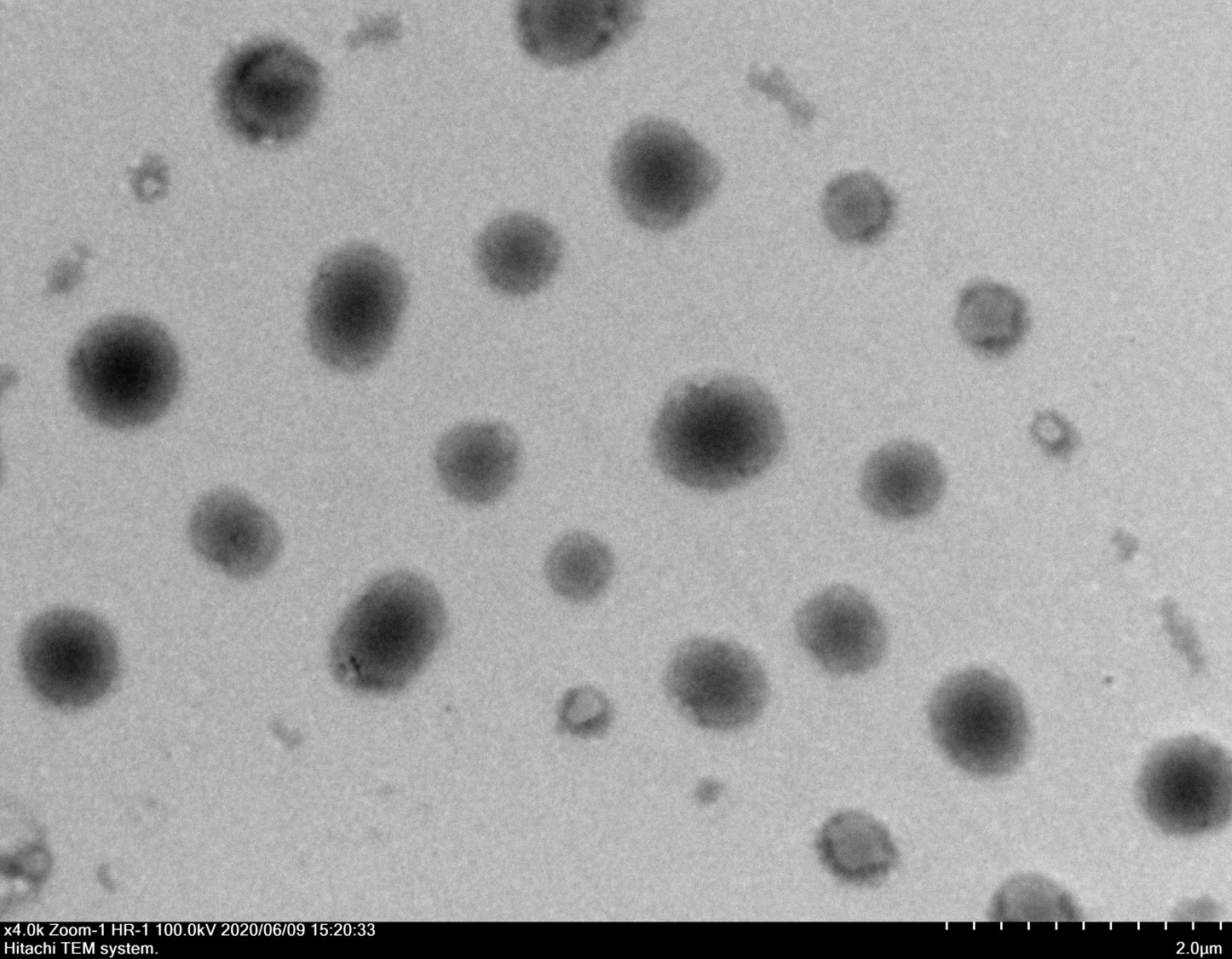

Supplement: Supplementary file 1 [file pharmaceutics-14-01134-s001.zip › pharmaceutics-1721084-supplementary/Supplementary/TEM/2.jpg]
